# Functional Amino Acids in Pigs and Chickens: Implication for Gut Health

**DOI:** 10.3389/fvets.2021.663727

**Published:** 2021-05-25

**Authors:** Tristan Chalvon-Demersay, Diana Luise, Nathalie Le Floc'h, Sophie Tesseraud, William Lambert, Paolo Bosi, Paolo Trevisi, Martin Beaumont, Etienne Corrent

**Affiliations:** ^1^METEX NOOVISTAGO, Paris, France; ^2^Department of Agricultural and Food Sciences, University of Bologna, Bologna, Italy; ^3^PEGASE, INRAE, Institut Agro, Saint Gilles, France; ^4^INRAE, Université de Tours, BOA, Nouzilly, France; ^5^GenPhySE, Université De Toulouse, INRAE, ENVT, Toulouse, France

**Keywords:** functional amino acids, oxidative stress, immunity, epithelial barrier, gut microbiota, weaning, coccidiosis

## Abstract

In pigs and broiler chickens, the gastrointestinal tract or gut is subjected to many challenges which alter performance, animal health, welfare and livability. Preventive strategies are needed to mitigate the impacts of these challenges on gut health while reducing the need to use antimicrobials. In the first part of the review, we propose a common definition of gut health for pig and chickens relying on four pillars, which correspond to the main functions of the digestive tract: (i) epithelial barrier and digestion, (ii) immune fitness, (iii) microbiota balance and (iv) oxidative stress homeostasis. For each pillar, we describe the most commonly associated indicators. In the second part of the review, we present the potential of functional amino acid supplementation to preserve and improve gut health in piglets and chickens. We highlight that amino acid supplementation strategies, based on their roles as precursors of energy and functional molecules, as signaling molecules and as microbiota modulators can positively contribute to gut health by supporting or restoring its four intertwined pillars. Additional work is still needed in order to determine the effective dose of supplementation and mode of administration that ensure the full benefits of amino acids. For this purpose, synergy between amino acids, effects of amino acid-derived metabolites and differences in the metabolic fate between free and protein-bound amino acids are research topics that need to be furtherly investigated.

## Introduction

The main functions of the gut are to ingest, digest, and absorb nutrients to support animal growth and physiological functions while protecting the organism against luminal harmful compounds (toxins, microorganisms, dietary antigens, etc.) (1). This dual function as a filter and a rampart explains the complexity of the organization of the digestive mucosa that is covered by a single layer of epithelial cells. The intestinal epithelium is constantly renewed by cell turnover mediated by stem cells located at the bottom of the crypts. During the migration along the crypt-villus axis, epithelial cells differentiate into absorptive (enterocytes) or secretory (goblet, paneth, enteroendocrine cells) lineages. This cellular complexity supports the two main functions of the intestinal epithelium: nutrition (e.g., secretion of digestive enzyme, nutrient absorption, hormone secretion) and barrier function (e.g., formation of tight junctions, secretion of antimicrobial peptides and mucus). Epithelial cells also communicate with immune cells located in the *lamina propria* that constitute a key component of the defensive system of the digestive mucosa, notably through the secretion of immunoglobulins. This complex organization of the digestive mucosa allows the establishment of a symbiotic relationship with the microbiota that colonizes the gut lumen. This consortium of bacteria, yeasts and protozoa collectively provide benefits to their animal host (2) notably through complex carbohydrate digestion, immune system tuning, and pathogens fighting (3, 4).

In livestock, both pigs and chickens are particularly subjected to digestive disturbances especially during the early life because of the immaturity of their digestive tract. Antimicrobial molecules have been massively used to control digestive diseases but increasing concerns on antibioresistance and environmental issues have urged to find non-antimicrobial disease control strategies. During the last decade, there was a significant reduction in the use of antimicrobial but further strategies are needed to maintain or improve the gut health status of pigs and poultry.

**Figure 1:**
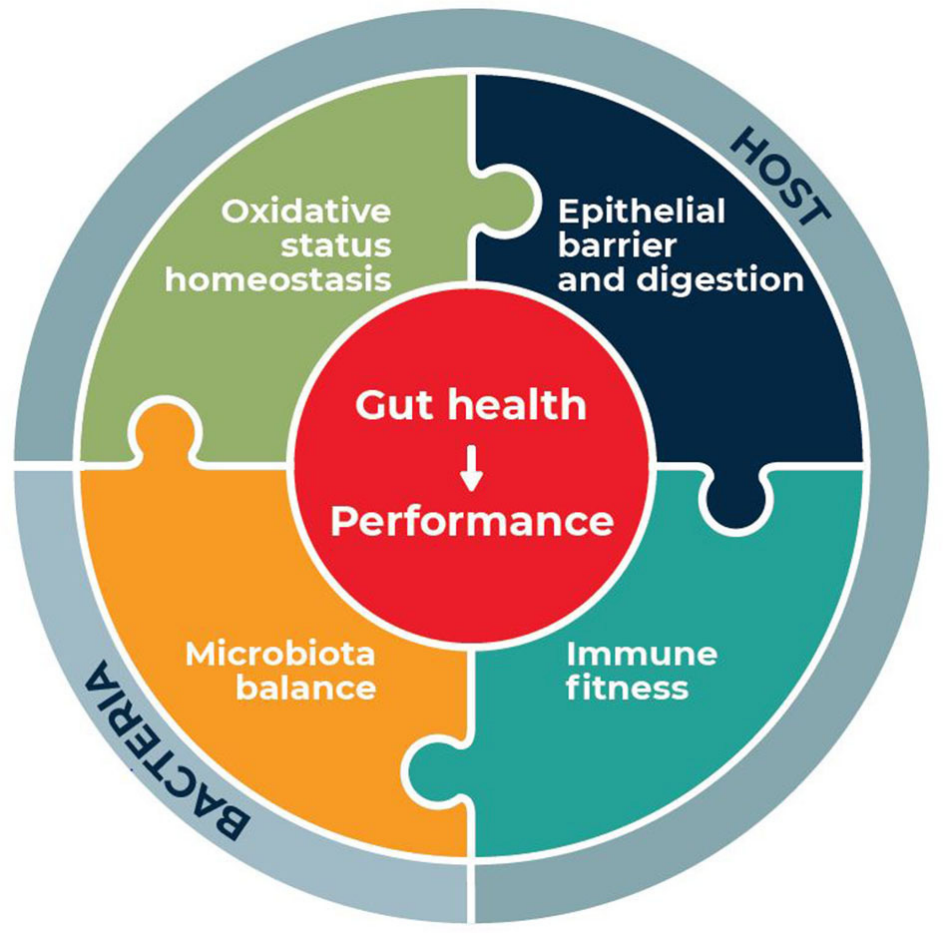
definition of gut health for farm animals.

Amino acids (AA) are major energy substrates in the intestinal mucosa, limiting constituents of key proteins of the gut barrier and they can regulate immune responses and oxidative stress (5). In this context, the aim of the present review is to summarize the potential of functional AA supplementation to preserve and restore gut health of pigs and chickens. Topic on which a large number of studies is available for both species. The first requirement to evaluate the effects of AA is a clear definition of gut health that could be applied to pigs and chickens and of its indicators (6) similarly to what has been done in humans (7). In line with the definition of gut health provided by Kogut and Arsenault (6) and Pluske et al. (1, 6), we consider that gut health provides resistance and resilience of the animals to respond and adapt to the challenges that they can encounter, allowing optimal performance, low mortality and morbidity and good overall health. According to our definition, gut health is characterized by four interconnected pillars: (1) epithelial barrier function and absorption (2) intestinal immune fitness (3) oxidative stress homeostasis and (4) microbiota balance as presented in Figure 1. Herein, we first detail the key components of these pillars and define related indicators in pigs and chickens. Then, we review the effects of dietary AA supplementation on gut health indicators providing, when available, a description of the potential mode of actions. Finally, we propose future directions of research to optimize the use of AA supplementation to ameliorate gut health in pigs and chickens.

## The Four Pillars of Gut Health and Associated Indicator

Pigs and chickens differ in terms of intestinal physiology and organization. To be able to generalize our definition of gut health to both species, we focus in this part on markers and indicators that are considered valid for both pigs and chickens.

### Epithelial Barrier, Digestion, and Nutrient Absorption

Function of digestion and absorption of the nutrients is realized through the coordinated actions of digestive enzymes and nutrient transporters. These digestion and absorption processes are directly related to the surface of the epithelium. This surface is a function of the height of the villus and the ratio between villus height and crypt depth which are key indicators of absorptive capacity and performance (8). Even a slight villus atrophy due to distress or illness can induce a consequent reduction of the digestive capacity by a reduction of the enzyme secretion from the apical part of the villi (9).

The mucus layer overlying the monolayer of intestinal epithelial cells is the first physical barrier of the gut. This mucus forms a gel and is composed by mucins which are glycoproteins secreted by goblet cells. It prevents the direct contact of toxins and pathogens with the epithelium (10). The number of goblet cells, the thickness of mucus layer as well as the level of expression of genes encoding for mucins are regarded as key indicators related of this barrier function (11, 12).

In addition, epithelial cell shedding (so called “anoikis”) and the fast renewal of the intestinal epithelium (3–5 days) is another mechanism providing protection against pathogens by limiting their adherence to epithelial cells. Rapid renewal of damaged enterocytes is supported by a high protein turnover and cell proliferation, and maintenance of functional enterocytes can therefore be considered as a marker of good intestinal function (13, 14). Low expression of caspase-3 in enterocytes indicates decreased apoptosis whereas increased proliferating cell nuclear antigen (protein which enhances DNA polymerase activity), mitotic index (number of cells undergoing mitosis divided by the total number of cells), and ornithine decarboxylase (protein involved in the first step of polyamine synthesis) activity are described as indicators of cellular proliferation (15).

This barrier function also relies on the sealing of epithelial cells which depends on the organization of tight junctions. Tight junctions, located at the apical side of the enterocytes, are multi protein complexes consisting of transmembrane proteins, such as occludin, claudins (claudin-1, claudin 2, claudin 3), tricellulin, and junctional adhesion molecules anchored with cytosolic molecules like zonula occludens proteins (ZO-1, ZO-2, and ZO-3). The abundance of these molecules is directly linked to a decrease in intestinal permeability (16). Disruption in gut barrier function increases gut permeability, occurrence of diarrhea and leaky gut syndrome.

In summary, the following parameters are considered good markers to monitor epithelial barrier and digestion: villous height, gene expression and/or protein levels of tight junctions, abundance of goblet cells or mucins, digestive enzyme activity, nutrient transporters, cell proliferation, diarrhea occurrence, intestinal permeability, cell apoptosis.

### Intestinal Immune Fitness

The physical barrier function of the gut is completed by innate and acquired immunity which constitutes two additional lines of defense. The gut is an important site of immunity in the body and can be subjected to inflammatory process. Inflammation activates immunity in order to fight against an infection and/or repair tissue damage. Inflammation is related to increased demand in energy and nutrients to synthesize cytokines and acute phase protein and activate the proliferation of the immune cells (17–19). Therefore, an excessive immune response, which could be defined as an imbalance between the level of inflammation and the challenge faced by the animal, can lead to an excessive and unnecessary use of energy and nutrients.

Immune fitness could be defined as “the capacity of the host immune system to respond in an appropriate manner to a challenge and to return or stay in immune homeostatic state in the case of the absence of a challenge.”

Low plasma circulating and/or low intestinal gut mucosa gene expression of proinflammatory cytokines (TNF-a, IFN-g, IL-1, IL-4, IL-6, IL-8) in the absence of a challenge and high secretion of immunoglobulins are indicators related to immune fitness. In addition, the intestinal concentration of secretory IgA which are a key component of mucosal defenses is an indicator of the adaptive immune response (20). Another key marker of immune fitness and inflammation is related to lymphocytes proliferation in the mucosa and their phenotype. For instance, high proportion of regulatory T cells in the gut mucosa (Tregs) expressing Foxp3 indicates an immunoregulatory phenotype (21).

In summary, the following parameters are considered good markers to monitor immune fitness: immunoglobulin concentrations, cytokines concentration, lymphocytes proliferation.

### Oxidative Stress Homeostasis

Oxidative stress occurs when the production of reactive oxygen species (ROS) such as superoxide is not balanced by the antioxidant defense (22). In that case, ROS may cause alteration of macromolecules including lipids (marked by increased malondialdehyde), proteins and DNA leading to cellular and tissue damages. The production of ROS is a physiological mechanism, these molecules being generated in the mitochondria during aerobic cellular metabolism (22). Besides, these molecules are synthesized by the cells of the innate immune system, like granulocytes and macrophages, and the epithelial cells to defend against pathogens (23). In the intestine, the main ROS generating enzymes are NOX1 and DUOX2 produced respectively by the epithelial cells and the neutrophils (24).

A tight control of ROS concentration is of primary importance and requires a delicate balance of systems involved in their generation and degradation. Oxidative stress homeostasis is defined as a “situation where the concentration of reactive oxygen is sufficient to transduce signal and counteract pathogens but insufficient to trigger cell damages to the host.”

Antioxidant system relies on the action of antioxidant molecules including vitamins such as vitamins E and C and metabolites like glutathione (GSH) in the oxidized form, and enzymes including superoxide dismutase (SOD), catalase (CAT), glutathione peroxidase (GSH-Px) and heme oxygenase (HO-1) which expression is under the control of the transcription factor Nuclear factor erythroid 2-related factor 2 (Nrf2) (25). Chaperone proteins such as HSP70 play also a role in the response to oxidative stress being involved in the removing of non-functional and potentially harmful proteins (26). Both the measurements of antioxidant molecules concentrations and antioxidant enzyme activities in the gut mucosa can be used as markers to assess oxidative status.

In summary, the following parameters are considered good markers to monitor oxidative stress at the gut level: total glutathione concentration, antioxidative enzyme expression or activity, antioxidative capacity, concentration of malondialdehyde, oxidized glutathione concentration.

### Microbiota Balance

In pigs and broilers, the intestine of healthy individuals is colonized by over than 500 species of bacteria, but also by fungi and protozoans (27, 28). Focusing on bacteria, for which the literature is more exhaustive, is a first approach to encompass microbiota complexity. In caecum and colon, microbial species belong essentially to the phyla *Firmicutes* (including *Clostridium, Enterococcus, Lactobacillus*, and *Ruminococcus* genera) and *Bacteroidetes* (including *Bacteroides* and *Prevotella* genera) (29, 30). These bacteria are in a homeostatic balance with the host and guarantee the protection of the gut. Indeed, commensal bacteria potentially prevent the overgrowth of pathogenic ones by competing for nutrients and adhesion sites and by synthesizing short chain fatty acids (SCFA) or antimicrobial peptides. The microbiota also plays a pivotal role to degrade the non-digestible compounds producing SCFA as source of energy as well as noxious compound like ammonia or polyamines when the substrates are indigestible proteins.

Microbial ecology is a new frontier for animal science and in some way mirrors what has been done in humans. Among the approaches proposed by Vangay et al. (31) to study the microbiota, the dysbiosis-centric view can fit with the livestock science. The authors defined four changes associated with dysbiosis: loss of keystone taxa, loss of biodiversity, blooms of pathogens and pathobionts and shift in functional capability. These changes can occur independently or altogether, which is most often the case (32). Microbiota balance could therefore be defined as the opposite of dysbiosis meaning “a bacterial population abundant and diverse with a high contribution of beneficial bacteria (Lactobacillus, Bifidobacterium, SCFA producers) at the expense of pathogenic ones” such as enterotoxigenic *Escherichia coli* (ETEC), *Campylobacter and Salmonella enteritidis*.

In summary, the following parameters are considered good markers to monitor microbiota: microbiota diversity, abundance of beneficial bacteria, abundance of parasites, abundance of harmful bacteria.

## Effect of Gut Health Challenges in Pigs and Broiler Chickens on Amino Acid Metabolism

### Early Weaning and Diarrhea in Piglets

In intensive pig farms, piglets are usually weaned between 2 and 5 weeks of age, when the gut and immune system are immature. Early weaning is a critical period during which they are separated from the sow and mixed with those of other litters in a new environment (33). These changes generate a stress which usually decreases water and feed intake and in turn affects negatively the gut health (33, 34). It was reported that piglets, in the immediate post-weaning, exhibit intestinal villous atrophy, crypt hyperplasia and lower number of goblet cells. Altered transepithelial resistance and depressed digestive enzymatic activities are also observed in association with immune cells infiltration, upregulation of pro-inflammatory cytokines and decrease in nutrient absorption (35). Intestinal oxidative stress indicators are also increased due to a reduction of the activity or gene expression of antioxidative enzymes (36). All these changes can decrease the ability of the host to digest and absorb nutrients (37) contribute to dysbiosis and translate into the occurrence of post-weaning diarrhea (38).

In addition, both protein content and protein synthesis in the gut increase after weaning indicating a high need to support the tissue development and adaptation to this period (39). Weaning is also believed to reshape AA metabolism especially at the gut level. Indeed, at weaning, the endogenous production of arginine is blunted in enterocytes which could lead to a shortage of arginine (40). Bacterial infection involving *E coli*, the major pathogenic bacterium involved in post-weaning diarrhea, is known to impair feed intake, modulate AA, gut and body metabolism because of the systemic inflammation induced by this infection. Two studies reported higher tryptophan requirement in piglets challenged with enterotoxigenic *E. coli* (41, 42). Accordingly, weaning under poor sanitary conditions induced a systemic inflammation that affected whole body tryptophan metabolism (43). Similar results were reported with threonine in piglets housed under challenged conditions and fed antibiotic-free diets (44–46). A recent study also revealed that immune stimulation induced by bacterial endotoxin (LPS) injection also led to an increase in the fluxes of GSH synthesis together with a decrease in plasma concentration of sulfur AA cysteine, one of the three AA constituting GSH, and methionine (47). Taking together, these results suggest that weaning may increase the need for AA and that weaned piglets could particularly benefit from increased supply of arginine, sulfur AA (SAA), tryptophan and threonine.

### Coccidiosis Challenge in Broiler Chickens

In broiler chickens, coccidiosis is the main challenge at the gut level, generating more than 3 billion dollars of loss every year in poultry industry (48). This challenge is caused by the infection of *Eimeria* species which colonize different parts of the intestinal tract. This coccidiosis decreases feed intake and growth and increases the susceptibility of necrotic enteritis leading to a further decrease in performance and increase in mortality (49).

The response of the host to the infection could be described in two phases: a damaging phase and a repairing and defense phase. As part of the damaging phase, coccidiosis is associated with decreased villus height, number of goblet cells, AA transporters and digestive enzymes leading to a lower AA availability as well as a decrease in transepithelial resistance and mucin expression (50). In addition, infection with any of the major *Eimeria* species leads to reduced plasma carotenoid thereby impairing protection against oxidative stress (51).

As part of the repairing and defense phase, it can be observed during coccidiosis an increase in inflammation as indicated by the increase of cytokines, nitric oxide and IgA production used to fight the parasites (50, 52). In addition, crypt cell proliferation occurs to replace damaged enterocytes and mucus production is enhanced to form a physical barrier against the pathogens (53). This latter effect could potentiate *Clostridium perfringens* colonization and necrotic enteritis as this bacteria can use intestinal mucus as a source of nutrients (49).

It has been reported that coccidiosis condition modulates AA metabolism. Indeed, it was shown that coccidiosis decreases the digestibility of nearly all AA (54). In the same study, Rochel et al. reported that coccidiosis-challenged birds had decreased plasma concentration of arginine, asparagine, glutamine, aspartate and increased concentration of ornithine, branched-chain AA (BCAA) and lysine. Interestingly, re-analysis of existing transcriptomic data from chicken cecal epithelia upon infection by *Eimeria tenella* (55) revealed that the expression of genes encoding for enzymes involved in threonine and arginine catabolism were increased during coccidiosis infection in the cecum (data not published) suggesting decreased availability of these amino acids for protein synthesis. To summarize, when broilers are facing coccidial or bacterial challenge, feed intake and digestibility of AA are reduced while they are showing a higher need for some of those functional AA, leading to an imbalance between supply and demand of AA in fast-growing broiler diets.

## Roles of Amino Acids in Intestinal Homeostasis

As suggested in the previous part, AA seem to have an important role in the maintenance of gut health as their demand can be increased during periods of challenge in both piglets and chickens. Based on the published literature on functional AA supplementation in piglets and broilers, we summarize below how AA could support and restore the four pillars of gut health previously described. By “support,” we refer to the way that AA can reinforce a pillar of gut health in the absence of challenge or prepare the function before a challenge. By “restore,” we refer to the way that AA can help the functions to recover and go back to homeostasis after a challenge like weaning in piglets and coccidiosis in broiler chickens. By “support and restore,” we refer to the way that AA prepare to a challenge and help the animal to recover. For each of the four pillars, a list of key indicators mentioned in the previous part was listed as shown in Tables 1, 2. The AA that influence these indicators related to the four pillars of gut health are listed in Table 1 by summarizing the evidence in piglets and in Table 2 by summarizing the evidence in broilers.

**Table 1:** Amino acids influencing the indicators related to the 4 pillars of gut health in piglets.

**Pillars**	**Epithelial barrier and digestion**			**Immune fitness**			**Oxidative stress homeostasis**			**Microbiota balance**		
Indicators of gut health	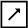 Villus height 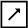 Tight junctions 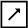 Goblet cells and mucins 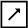 Digestive enzymes activity 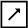 Transporters 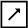 Cell proliferation	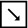 Diarrhea 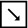 Permeability 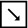 Cell apoptosis		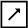 Immunoglobulins 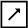 Anti-inflammatory cytokines	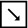 Pro-inflammatory cytokines 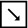 Lymphocytes proliferation		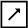 Total Glutathione 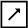 Antioxidative enzymes 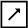 Anti-oxidative capacity	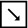 Malondialdehyde 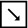 Oxidized glutathione		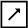 Diversity 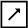 Beneficial bacteria (Lactobacillus, Bifidobacterium)	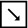 Parasites (Eimeria) 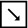 Harmful bacteria (Enterobacteria, Clostridium, Campylobacter)	
**Effect of amino acids**	**Support**	**Support and restore**	**Restore**	**Support**	**Support and restore**	**Restore**	**Support**	**Support and restore**	**Restore**	**Support**	**Support and restore**	**Restore**
Asparagine	(56)	(57)		(56)								
Aspartate		(58)		(59, 60)				(61)		(59)		
Arginine	(62–67)	(68)	(69)	(62, 64)	(68)		(62, 65, 70, 71)					
Cysteine	(72)	(73)		(72)	(73)		(72)					
Glutamate or monosodium Glutamate	(67, 74–77)	(78)	(61)	(79)	(75, 78)		(77)	(78)	(61)			
Glutamine	(62, 80–89)	(90–93)	(94)	(62, 88)	(92, 93, 95)	(94, 96)	(88, 97)	(92)				
Isoleucine	(98)	(99)			(99, 100)							
Leucine		(101)										
Lysine	(102)	(103)								(102)		
Methionine	(104, 105)						(105)					
Proline	(106)											
Serine	(107)			(107)			(107)					
Threonine	(108, 109)			(110)								
Tryptophan	(111)	(42, 112–114)		(111)	(113, 115)		(116)	(114)				

*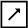, increased in response to AA supplementation; 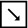, decreased in response to AA supplementation*.

**Table 2:** Amino acids influencing the indicators related to the 4 pillars of gut health in broiler chickens.

**Pillars**	**Epithelial barrier and digestion**			**Immune fitness**			**Oxidative stress homeostasis**			**Microbiota balance**		
Indicators of gut health	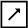 Villus height 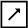 Tight junctions 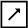 Goblet cells and mucins 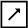 Digestive enzymes activity 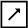 Transporters 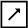 Cell proliferation	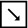 Diarrhea 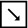 Permeability 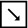 Cell apoptosis		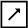 Immunoglobulins 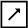 Anti-inflammatory cytokines	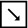 Pro-inflammatory Cytokines 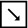 Lymphocytes proliferation		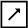 Total Glutathione 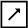 Antioxidative enzymes 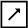 Anti-oxidative capacity	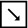 Malondialdehyde 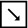 Oxidized glutathione		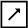 Diversity 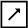 Beneficial bacteria (eg. Lactobacillus, Bifidobacterium)	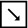 Parasites (Eimeria) 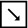 Harmful bacteria (E. coli, Enterobacteria, Clostridium, Campylobacter)	
**Effect of amino acids**	**Support**	**Support and restore**	**Restore**	**Support**	**Support and restore**	**Restore**	**Support**	**Support and restore**	**Restore**	**Support**	**Support and restore**	**Restore**
Arginine	(50, 117, 118)			(119–121)	(50, 122, 123)						(52, 117, 118)	
Glutamate		(124, 125)										
Glutamine	(125, 126)	(125, 127–129)		(125)	(125)			(127)				
Glycine	(130)											
Lysine								(131)				
Methionine					(132)			(131)				
Threonine	(130, 133)	(134, 135)		(133)	(134)	(133)	(131)			(133)		
Tryptophan					(122)		(136)			(136)		

*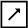, increased in response to AA supplementation; 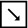, decreased in response to AA supplementation*.

These two tables suggest that investigations about AA effects on gut health are scarcer in broilers than piglets. Most broiler studies are focusing on arginine, glutamine and threonine, while piglet studies are investigating a broader scope of AA. It is difficult to clearly identify different effects of AA supplementation across the two species while it is known that AA requirements and metabolism might differ in pigs and chickens. Furthermore, it is interesting to note that in both the species most studies tested very high doses of supplementation of AA (between 0.5 and 1.0% as-fed basis) which lead to level of AA far above the recommendation for growth in pigs and chickens. This could be due to the fact that studies aimed to reveal the functional properties of amino acids.

It is interesting to note that some AA, especially aspartate, arginine, cysteine, glutamate or mono sodium glutamate (MSG), and glutamine for piglets and arginine, glutamine, threonine and tryptophan for broilers are involved in three out of the four pillars of gut health confirming two main aspects: (1) these pillars are strongly interconnected and interdependent; (2) AA have different functions and can modulate several metabolic pathways and functions depending to the specific conditions.

These functional properties have been well-described in the literature and rely on the following functional properties of AA: (1) AA are energy sources and precursors of functional molecules and proteins, (2) AA modulate gene expression and protein phosphorylation and finally, (3) AA can serve as microbiota modulators.

### Amino Acids Are Energy Sources and Precursors of Functional Molecules and Proteins

Several AA can serve as a source of energy for the gut epithelium and are therefore considered to favor gut development and epithelial barrier. Indeed, it has been reported that most of dietary glutamine and glutamate (>90%) after conversion into α-ketoglutarate fuel the Krebs cycle and are used as a source of energy by enterocytes (137). Accordingly, in piglets, the supplementation of feed with asparagine, aspartate, glutamine, alanyl-glutamine and MSG are associated with an increase of energy availability in the intestine as shown by higher intestinal levels of ATP, adenylate energy charge (AEC) and lower AMP:ATP ratio (57, 58, 77, 92). Similarly, the roles of glutamate and glutamine as substrate for ATP production has been reported *in vitro* using chicken enterocytes, glutamate being the most potent source of energy (138).

The importance of AA for gut health also relies on the abundance of particular AAs in functional proteins. For example, threonine is critical for epithelial barrier function being the most abundant indispensable AA in mucins (53). Finally, AAs are also pivotal for gut health as precursors of functional molecules. For example, glycine, glutamate and cysteine are the three AAs composing glutathione (GSH), a tripeptide synthesized in the cytosol that play a key role in the regulation of the oxidative stress through its scavenging effect on free radicals (72). The concentrations in glutathione in the small intestine (jejunum and ileum) was decreased by 50% in piglets fed a SAA-free diet compared to those fed a well-balanced diet (139).

### Amino Acids Can Modulate Gene Expression and Protein Phosphorylation

In addition to being precursors of energy and functional molecules and proteins, AAs are signaling molecules; their abundance in cells directly modulates some metabolic pathways by modifying gene expression and protein phosphorylation. In piglets, leucine and glutamate supplementations in feed *in vivo* were able to increase the level of phosphorylation of mTOR, a major regulator of protein synthesis, and some of its downstream targets (4-EBP1, S6K) in the different parts of the gut (75, 78, 101, 140). In line with these results, Corl et al. (141) reported that arginine and BCAA increased the phosphorylation level of p70S6k, a downstream target of mTOR, in rotavirus-infected piglets' jejunal segments. Similarly, increasing the glycine level triggers cell proliferation, protein synthesis, phosphorylation of mTOR, 4EBP-1 and p70S6K in intestinal porcine IPEC-1 cells (142). In broiler chickens, evidence is scarce but a key role of arginine as a regulator of protein synthesis in the gut is suggested. Indeed, Tan et al. reported that supplemental dietary arginine attenuates intestinal mucosal disruption in broiler chickens during a coccidial vaccine challenge through an increase of mRNA expression of jejunal genes related to kinase activity, such as *mTOR, Raptor* and *RP6KB1* (50). The effect of AA supplementation in feed on gene expression is well-described. For example, the expression of AA and glucose transporters responds to AA supplementation, particularly to branched-chain amino acids and lysine in piglets (103) and to lysine, methionine and threonine in broiler (143) suggesting that AA supplementation could mitigate the effects of challenge and support gut health by improving nutrient absorption (144). Supplementation of glutamine to weanling diet can promote the expression of genes related with the reduction of oxidative stress (88). Similarly, SAAs can control *Nrf2* expression in the liver, a transcription factor controlling the expression of antioxidant redox buffering enzymes and the production of other scavenging systems for reactive oxygen species like methionine sulphoxide reductases (145).

### Amino Acids Are Microbiota Modulators

*In vitro* studies, based on single strains and mixed community derived by intestinal content of piglets have shown that AAs can contribute in influencing the metabolism and the development of bacteria (146–148). This suggests that AAs can regulate the gut microbiota composition and activity. This microbiota-modulating effect of AAs has already been investigated with a main focus on tryptophan and arginine.

Indeed, in weaned pigs, 0.4% tryptophan supplementation for 4 weeks increased *Lactobacillus* and *Clostridium XI* in the jejunum (111). Alpha diversity indices were enhanced in response to tryptophan supplementation in both weaned piglets (111) and fattening pigs susceptible to intestinal adhesion of ETEC F4 (149). 1.0% arginine supplementation for 60 days in fattening pigs increased *Canobacteria* and in combination with 1.0% Leucine (Leu) it increased *Bacteriodes* and reduced *Clostridium* sensu stricto, *Terrisporobacter* and Escherichia-Shigella in the colon (150). In sows, arginine supplementation increased both the *Bacteroidaceae* family and the *Bacteroides* genus in feces (151).

In broilers, it has been reported that arginine supplementation can alleviate gut injury and normalize the ileal microbiota of *C. perfringens*-challenged chickens (117). Furthermore, in broiler chickens facing a 2 h-transportation stress, tryptophan supplementation increased the population of beneficial bacteria (Enterococci, Bifidobacteria and Lactobacilli) and reduced the population of pathogenic ones (Clostridia, Enterobacteria and Campylobacter) in the cecum digesta suggesting a positive effect of this AA on microbial balance (136). Similarly, a total SAAs supply exerted a beneficial effect in broiler cecal microbial community by increasing the alpha diversity of the microbiota and by promoting the microbial metabolisms related to carbohydrate, AA, nucleotide, and lipid (152).

The metabolism of AA by the gut microbiota releases numerous metabolites in the intestinal lumen (153). These bioactive compounds are key molecular intermediates between the microbiota and its host. Similarly to carbohydrates, AA can serve as precursors for the production of the main SCFA including acetate and butyrate that are well-known regulators of gut health (154). Moreover, some SCFA are produced exclusively from AA (isobutyrate, isovalerate, 2-methylbutyrate) but their effect on gut health has not been extensively studied (153). Recent research has highlighted the beneficial role on gut health of bacterial metabolites derived from tryptophan (indolic compounds) (155). Catabolism of AA by the gut microbiota also produces amines and polyamines such as putrescine, cadaverine and 5-aminovalerate. The effects on gut health of these metabolites are not clear yet since both beneficial and detrimental effects were described according to the studies and concentration tested (153). It is also worth noting that AA degradation by the microbiota can release toxic compounds such as deamination-derived ammonia, cysteine-derived hydrogen sulfide and tyrosine derived p-cresol (153). In Table 3, we summarized the main metabolites yielded by bacterial metabolism of the AA that were tested in the *in vivo* trial that included in the present review. We also listed the direct effects of these AA-derived metabolites on gut health. Overall, it is clear that metabolites derived from the s yielded by bacterial catabolism of the AA can mediate part of the action of AA on the four pillar of gut health and more work is needed to validate this hypothesis in pigs and poultry.

**Table 3:** Main metabolites produced by amino acid metabolism by the in the gut microbiota and associated effect on gut health.

**AA**	**AA derived metabolites with effect on gut health**	**Effects on gut health**	**Reference**
**Arginine**	Putrescine	Involved in cell proliferation	(153, 156)
	Spermine and spermidine	Involved in DNA and protein syntheses	(153, 156)
**Asparagine**	Converted to Aspartate		(157)
**Aspartate**	Acetate	Is a precursor for fatty acid synthesis and an energy source	(158, 159)
**Cysteine**	H_2_S	Is a source of energy for colonocytes in low concentration Inhibits mitochondrial respiration and SCFA oxidation, disrupts mucus layer in high concentration	(153, 157, 160)
	Acetate	Is a precursor for fatty acid synthesis and an energy source	(157, 158)
	Butyrate	Is a major energy source for colonocytes	(157, 159)
**Glutamate**	Acetate	Is a precursor for fatty acid synthesis and an energy source	(157, 158)
	Butyrate	Is a major energy source for colonocytes	(157, 159)
**Glutamine**	Converted to Glutamate		(157)
**Glycine**	Acetate	Is a precursor for fatty acid synthesis and an energy source	(157, 158)
**Isoleucine**	2-Methylbutyrate or converted to Valine	Its effect is poorly documented	(153, 157)
**Leucine**	Isovalerate	Inhibits tight junction protein destabilization together with isobutyrate	(153, 161)
	Acetate	Is a precursor for fatty acid synthesis and an energy source	(158)
	Butyrate	Is a major energy source for colonocytes	(159)
**Lysine**	Acetate	Is a precursor for fatty acid synthesis and an energy source	(157, 158)
	Butyrate	Is a major energy source for colonocytes	(157, 159)
	Cadaverine	Can be toxic at high dose	(153, 157)
	5-aminovalerate	Can be toxic at high dose	(153)
**Serine**	Butyrate	Is a major energy source for colonocytes	(157, 159)
**Methionine**	Butyrate	Is a major energy source for colonocytes	(157, 159)
**Proline**	Acetate	Is a precursor for fatty acid synthesis and an energy source	(157, 158)
**Threonine**	Butyrate	Is a major energy source for colonocytes	(157, 159)
	Acetate	Is a precursor for fatty acid synthesis and an energy source	(157, 158)
**Tryptophan**	Indole	Increases the gene expression of tight junctions Reduces the expression of proinflammatory cytokines and chemokines while inducing the expression of anti-inflammatory cytokines	(155, 162)
	Phenol	Increases permeability *in vitro*	(163)
	Serotonin (5-HIAA)	Involves in the modulation of the gut immune system	(156)
	Tryptamine	Regulates intestinal motility and immune function	(157, 164)

## Concluding Remarks

This work confirms that supplementation of free AA, based on their roles as precursors of energy and functional molecules, as signaling molecules and as microbiota modulators, can contribute to gut health of monogastric animals by supporting or restoring its four intertwined pillars. The fact that piglet and broiler gut health positively benefit from AA supplementation indicates that under challenging conditions, those AA may become indispensable for optimal performance and health. Additional work is still needed in order to take the full benefits of AA functions while decreasing the effective dose of supplementation. For this purpose, synergy between AA, effects of AA derived metabolites, difference in the metabolic fate between free and protein-bound AA are research topics that need to be furtherly investigated.

## Author Contributions

TC-D, WL, and EC contributed to the conception and structure of the paper. TC-D organized the literature review and wrote the first draft of the paper. TC-D, DL, NL, PB, PT, MB, and ST contributed to manuscript writing and revision. All authors approved the final version for submission.

## Conflict of Interest

The authors declare that the research was conducted in the absence of any commercial or financial relationships that could be construed as a potential conflict of interest.
